# Application of Artificial Neural Network in miRNA Biomarker Selection and Precise Diagnosis of Colorectal Cancer

**DOI:** 10.29252/.23.3.175

**Published:** 2019-05

**Authors:** Saeid Afshar, Sepideh Afshar, Emily Warden, Hamed Manochehri, Massoud Saidijam

**Affiliations:** 1 Research Center for Molecular Medicine, Hamadan University of Medical Science, Hamadan, Iran; 2 Wellman Center for Photomedicine, Massachusetts General Hospital, Harvard Medical School, Boston, Massachusetts 02114, USA

**Keywords:** Artificial neural network, Biomarkers, Colorectal neoplasms, Diagnosis, MicroRNAs

## Abstract

**Background:**

The early diagnosis of colorectal cancer (CRC) is associated with improved survival rates, and development of novel non-invasive, sensitive, and specific diagnostic tests is highly demanded. The objective of this paper was to identify commonly circulating microRNA (miRNA) biomarkers for use in CRC diagnosis.

**Methods:**

An artificial neural network (ANN) model was proposed in this work. Among miRNAs retrieved from the Gene Expression Omnibus dataset, four miRNAs with the best miRNA score were selected by ANN units.

**Results:**

The simulation results showed that the designed ANN model could accurately classify the sample data into cancerous or non-cancerous. Furthermore, based on the results of evaluated ANN model, the area under the ROC curve (AUC) of the designed ANN model as well as the regression coefficient between the output of the ANN and the expected output was one. The confusion matrix of the ANN model indicated that all non-cancerous patients were predicted as normal, and the cancerous patients as cancerous.

**Conclusion:**

Our findings suggest that the improved model can be used as a robust prediction toolbox for cancer diagnosis. In conclusion, by using ANN, circulatory miRNAs can be used as a non-invasive, sensitive and specific diagnostic marker.

## INTRODUCTION

Colorectal cancer (CRC) is the third most common cancer globally, accounting for half a million deaths per annum. The underlying pathogenesis of CRC involves uncontrolled division of bowel epithelium[1-3]. Since early diagnosis of CRC is associated with improved survival rate, screening methods such as colonoscopy and CT-colonoscopy are recommended within certain patient populations[4,5]. Challenges to non-invasive screening tests such as carcinoembryonic antigen, carbohydrate antigen 19-9, and fecal occult blood test include low specificity and sensitivity. Thus, the development of a novel non-invasive, sensitive and specific test for CRC screening using protein, DNA, or RNA biomarkers, found in blood or stool, may be desirable[6,7].

MicroRNAs (miRNAs) represent a potentially appropriate biomarker for use in non-invasive diagnostic and screening[8,9]. Similar to small non-coding RNAs, miRNAs consist of approximately 20-24 nucleotides capable of regulating a range of cellular processes, including proliferation, differentiation, and apoptosis[10]. MiRNAs down-regulate their target genes post-transcriptionally via binding to 3’ untranslated regions of target mRNA. Importantly, dysregulation of miRNA expression due to epigenetic factors is characteristic of CRC[2,11], and according to recent studies, there is a stable and reproducible level of circulatory miRNAs[12]. Hence, a particular miRNA profile detected in plasma or serum may be used for the purpose of non-invasive CRC screening. MiRNA profiles depend greatly on different biological processes; as a result, a nonlinear relationship exists between the miRNA expressions and the presence of malignancy. Due to this nonlinearity, cancer diagnostics based on miRNA microarray using traditional linear models is both problematic and impractical[13].

At present, an empirical model is needed in order to correlate the diagnosis of CRC with specific miRNA profiles. Inspired by the central nervous system of animals, the artificial neural network (ANN) is often used to solve massive computational processes. The ANN consists of simple elements known as neurons, which are connected by weight coefficients. Upon training the ANN and adjusting weight matrices, a particular result can be driven from specific independent variables[14-16]. The main training approaches for the structured ANN can be classified into supervised and unsupervised techniques. In contrast to the unsupervised method, the supervised learning method is performed via a training process in which input data are categorized depending on output values[17,18]. Due to the high performance in modeling a nonlinear correlation between independent variables and their corresponding outcome, ANN is used in a broad range of cancer-related applications, including image processing, prognosis prediction, screening, diagnosis, and response to treatment[16,19-21]. The use of microarray miRNA profiles for application in cancer diagnostics by means of conventional, statistical linear models is at present unreliable. The purpose of this paper was to identify circulatory miRNA profiles using ANN, thereby building an accurate nonlinear model to link miRNA profile with CRC diagnosis.

## MATERIALS AND METHODS

The purpose of this paper was to use ANN as an efficient tool for diagnosis of CRC, given some miRNAs expression measurement. In order to achieve this end, dominant miRNAs expression was first identified. Among different miRNAs expression levels observed in CRC and healthy control samples, extracted from the dataset for pancreatic and biliary tract cancers, the prominent miRNAs were selected based on their signal-to-noise (S/N) ratio. The number of utilized indicative miRNAs expression in our diagnostic technique was further reduced by designing ANN units. Finally, the nominated miRNAs expressions were used as the input to a multilayer perception ANN model with three layers constructed and trained to predict the cancer diagnosis.

### MiRNA expression profile dataset

The miRNA expression dataset of pancreatic and biliary tract cancers was downloaded from Gene Expression Omnibus (GEO) database (http://www.ncbi. nlm.nih.gov/geo/) under the accession number GSE59856[22]. This dataset contains the miRNA expression profile of 571 serum samples including fifty patients with CRC, 150 healthy controls, and 371 patients with other digestive tract cancers. In this study, the miRNAs expression of 200 samples including 150 healthy control and fifty patients with CRC were selected for more analysis and validation of the ANN model.

### Selection of miRNAs by highest score

Initially, the levels of expression of 2555 miRNAs were ranked based on the S/N as follows:


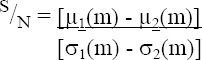


where µ and σ denote the means and standard deviations of miRNAs expression in samples from cancerous and healthy control categories, respectively[23].

MiRNAs with S/N >2.5 were selected for further analysis, while other miRNAs were excluded. In order to reduce the number of miRNAs in the learning process, miRNAs scores calculated by the ANN units were used[24]. These ANN units are composed of 10 neurons as the hidden layer and one neuron as the output layer, built in the neural network pattern recognition toolbox of MATLAB 2013b. Next, each miRNA expression value was normalized between 0 and 1, and the dataset with 200 samples was randomly classified into the training dataset (70%), validating dataset (15%), and test dataset (15%). The nonparametric correlation of the output value of every trained ANN with diagnosis status was evaluated using SPSS 16 (IBM Corporation, Arnmonk, NY, USA). These correlation coefficients were applied for both final ranking of miRNAs as well as the selection of a minimum number of miRNAs for the analysis by ANN; the correlation coefficient more than 0.7 and *p* value less than 0.01 were selected as a cut-off point.

### ANN model architecture and evaluation

A multilayer perceptron ANN model with three layers was created in MATLAB. The input layer included four neurons related to four selected miRNAs as the input data, and the hidden layer consisted of seven neurons transforming the input data to the output layer containing a single neuron. The values of output layer were 0 and 1, categorized as healthy control or cancerous, respectively. To minimize the number of input parameters used in the training process, the ANN performance was examined using multiple miRNAs sets, each containing different numbers of miRNAs.

Initially, the ANN was trained using the miRNA with the highest miRNA score as the input, thereby enabling the performance of the ANN model to be examined. Subsequently, the miRNA with the second highest score was added, and the performance examination was repeated. Until best performance was achieved, this process was continued.

After the miRNA expression values were normalized between 0 and 1, with mapminmax function and randomized division of the input data into the training dataset (70%), validating dataset (15%), and test dataset (15%), the training process was performed using the Levenberg-Marquardt learning function[21,25] with learning rate of 0.1. Finally, to evaluate the ANN model, the area under receiver operating characteristic (ROC) curve (AUC), confusion matrix, and linear regression were plotted. The ROC curve is the plot of sensitivity (true positive rate) against 1-specificity (false positive rate) and was created by SPSS version 16.0. The confusion matrix, also known as the error matrix, comprises a visualization of the percentages of the correct and incorrect classifications. A linear regression model was used to investigate the relationship between the output of the trained ANN model and the expected output. The confusion matrix and the linear regression plot were created in MATLAB.

### Prediction of target genes for each selected miRNA

As mentioned above, a number of miRNA expression level was utilized in our diagnosis technique. It is understood that there is a correlation between the target genes of these miRNAs and the signaling pathway of the CRC[10,13]. The target genes of the hsa-miR-6726-5p, the hsa-miR-7111-5p, the hsa-miR-1247-3p, and the hsa-miR-614 can be predicted via two well-known online prediction software, TargetScan (http://www.targetscan.org) and MRMicroT-CDS (http://diana.imis.athena-innovation.gr/DianaTools/index.php?r=mrmicrot/index). For each miRNA, one target gene, which has the highest score and has a role in cancer-related biological function including proliferation and survival signaling pathway, was selected.

## RESULTS

### Selection of miRNAs biomarkers

**Fig.1:**
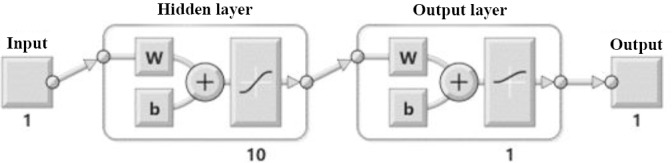
ANN for ranking miRNA with 10 hidden layers and one output layer. w, weights; b: bias

**Fig. 2:**
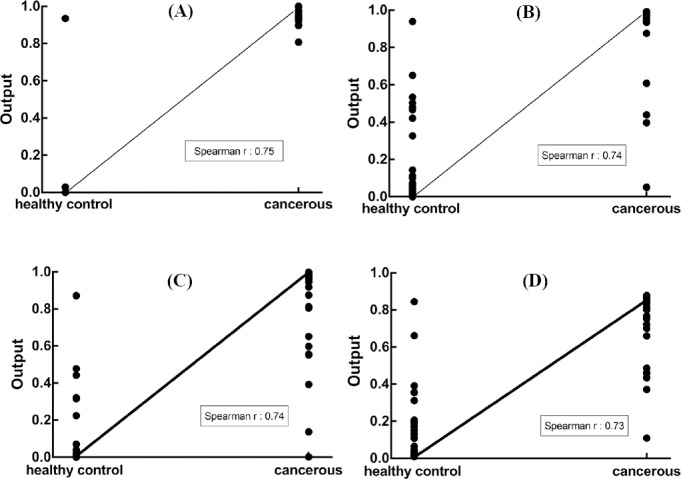
Correlation of output value of each ANN unit with diagnosis status for four top score miRNAs. (a) hsa-miR-6726-5p; (b) hsa-miR-7111-5p; (c) hsa-miR-1247-3p; (d) hsa-miR-614.

The first step of simulations corresponds to selecting the dominant miRNAs. According to the S/N threshold, S/N > 2.5, 459 miRNAs expression data were selected from the GEO database (http://www.ncbi.nlm.nih.gov/geo/). In this paper, 200 serum samples with normalized miRNAs expressions were used. The miRNAs were assigned with their scores defined by the ANN units, which has 10 hidden layers and one output layer (Fig. 1). The scored miRNAs using the designed ANN were ranked based on their scores. The top four selected miRNAs and their corresponding scores are shown in Table 1. These miRNAs are hsa-miR-6726-5p, hsa-miR-7111-5p, hsa- miR-1247-3p, and hsa-miR-614. The graph of correlation of output value of each ANN unit with diagnosis status for four top score miRNAs is shown in Figure 2. It is observed from this Figure that the four selected miRNA expressions selected based on their highest assigned score were adequate to achieve a good performance with a minimum number of biomarkers for the training process.

**Table 1:** Top four miRNAs selected based on the miRNA score ranking

miRNA	miRBase accession number	miRNA score^a^	S/N	Cumulative mean squared error^b^
hsa-miR-6726-5p	MIMAT0027353	0.75	83.84	5.30 × 10^-2^
hsa-miR-7111-5p	MIMAT0028119	0.74	18.87	5.11 × 10^-3^
hsa-miR-1247-3p	MIMAT0022721	0.74	21.56	7.76 × 10^-4^
hsa-miR-614	MIMAT0030429	0.73	9.52	1.06 × 10^-5^

a miRNA score is equal to Spearman’s correlation coefficient between output and the expected output of each ANN unit;

b mean square error of training process of final ANN for different numbers of miRNA as an input. Initially, the performance of ANN for input with the first miRNA is calculated, and by adding next miRNA to input at each step, performance of ANN is calculated.

### Construction of ANN model

**Fig. 3:**
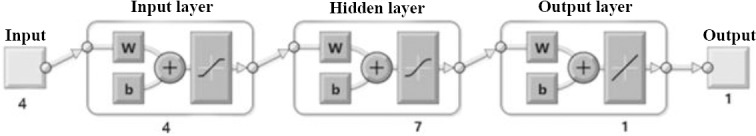
Final ANN model with 4.7.1 architecture created for the training process. This model consists of an input layer with four neurons, a hidden layer with seven neurons, and an output layer with one neuron. Transforming function of the input layer is satin, transforming function of the hidden layer is tansig, and transforming function of the output layer is purelin. w, weights; b: bias

**Fig. 4:**
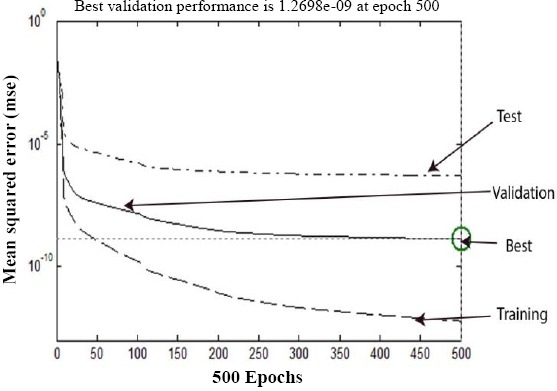
The plot of performance in log scale against epoch number. Performance for each of the training, validation, and test sets rapidly decreased as the network was trained.

In the next step, ANN was designed for the diagnosis purpose. The chosen ANN model was a 4-7-1 network framework consisting of an input layer with “satlins” transforming functions, a hidden layer with “tansig” transforming functions, and an output layer with “purelin” transforming functions (Fig. 3). The input variables include the normalized miRNA expression values defined in the previous step, listed as hsa-miR-6726-5p, hsa-miR-7111-5p, hsa-miR-1247-3p, and hsa-miR-614. The performance of the ANN is shown in Figure 4. This Figure shows that the best validation performance, the output mean squared error, of the trained ANN model was reached at epoch 500 and was around 1.27 × 10^-9^. At this point, the output of the model, which was 0 and 1, was assigned as non-cancerous and cancerous patients, respectively, can be used for the diagnosis.

### Evaluation of ANN model

**Fig. 5:**
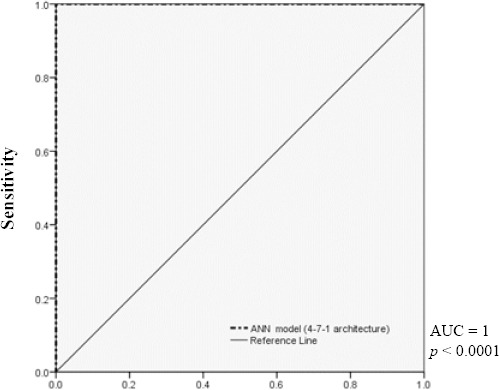
The ROC curve of trained final ANN model with 4-7-1 architecture. The area under the ROC curve (AUC) was higher than that of others and was 1.

**Fig. 6:**
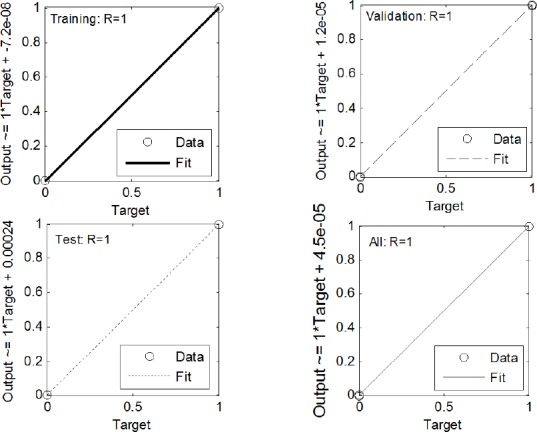
Regression plots for training, validating, test, and total data set. R values for all plots are equal to one.

**Fig. 7:**
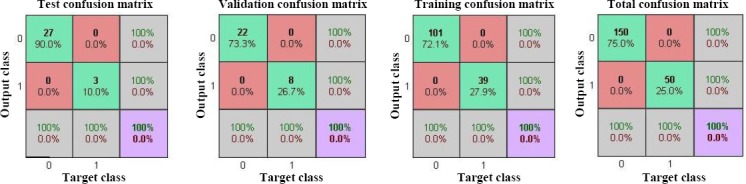
Confusion matrix showing the percentages of correct and incorrect classifications for training, validating, test and total dataset. Correct classifications are the green squares and incorrect classifications are the red squares. Confusion matrices for all dataset show the 100% correct classification by ANN.

The efficiency of the trained ANN model can be analyzed by studying the ROC curve, which is exhibited in Figure 5. According to this Figure, AUC of the designed ANN model was 1. The result of the ROC curve and AUC indicated that this ANN model accurately predicts the expected results. Furthermore, a linear regression plot was utilized to validate the performance of the trained ANN model depicted in Figure 6. As seen in Figure 6, the regression coefficient between the output of the ANN and the expected output was 1. Therefore, the ANN model could accurately predict the expected output, and there was an exact linear relationship between the predicted output and the expected output. Finally, in order to validate the accuracy of the classification, the confusion matrix was constructed and sketched in Figure 7. According to the results of the total confusion matrix of the ANN model, 75% of all datasets, which was not cancerous patients, predicted as normal, and 25%, which was cancerous patients, predicted as cancerous.

### Target genes of miRNAs

It was quoted before that the target genes of every miRNA expression play an important role in the signaling pathway of cancer. Among different predicted target genes of every miRNA, the most dominant one was selected based on a prediction score and the gene’s role in cancer-related biological function, including proliferation and survival signaling pathway. As depicted in Table 2, mitogen-activated protein kinase 1(MAPK1), ras homolog family member T1 (RHOT1), BCL2-antagonist/killer 1 (Bak1), and Bcl-2-related ovarian killer (BOK) were selected as target genes of the hsa-miR-6726-3p, the hsa-miR-614, the hsa-miR-7111-5p, and the hsa-miR-1247-3p, respectively.

**Table 2:** miRNAs target prediction with two online tools

MiRNA	Target scan		Diana MRMicroT score

	(Total context ++ score)	Site counts	
hsa-miR-6726-3p/ MAPK1	-0.2	One 7mer and one 6mer	0.37
hsa-miR-614 /RHOT1	-0.52	One 8mer	0.49
hsa-miR-7111-5p /BAK1	-0.9	One 8mer, two 7mer, and one 6mer	0.47
hsa-miR-1247-3p/BOK	-1.57	Six 7mer and one 6mer	0.51

## DISCUSSIONS

Early stage diagnosis of CRC, a malignancy with a high mortality rate, may lead to improved patient survival. While colonoscopy remains the gold standard screening tool for CRC, the procedure has several shortcomings, including invasiveness and patient discomfort[26]. On the other hand, non-invasive tests such as fecal occult blood test and carcinoembryonic antigen suffer from low sensitivity and specificity[27,28].

A previous study has indicated that miRNAs are potential candidates for use in early cancer detection[29]. Accordingly, examination of expression levels of a panel of circulatory miRNA could be used to classify patients into cancerous or healthy groups. In the present study, an ANN model provided a good predictive accuracy while minimizing the number of biomarkers needed for accurate classification. We hypothesize that this technique has the potential to increase the accuracy of diagnostic CRC testing.

In this paper, the classification of sample data obtained from GEO database into cancerous and healthy control was attempted. Four miRNAs, i.e. miR-1247-3p, miR-614, miR-6726-5p, and miR-7111-5p, were shown to be optimal for the accurate diagnosis of CRC using ANN units. It was also observed from the GEO dataset that the expressions of the miR-1247-3p and miR-7111-5p in the CRC patients were higher than the healthy control, but those of miR-614 and miR-6726-5p were lower compared to the healthy control.

The simulation results, the results of confusion matrices and the regression plot, indicated an adequate performance provided by the modeled ANN.

As evidenced in the literature, miR-1247-3p is correlated with metastasis in hepatocellular carcinoma[30]. The biological function of miR-6275-5p, miR-7111-5p, and miR-614 is, however, not clearly identified. The results of miRNA target prediction indicate that the MAPK1, RHOT1, Bak1, and BOK are the most important target genes for miR-6726-3p, miR-614, miR-7111-5p, and the miR-1247-3p, respectively. According to Wei *et al*.[31], down-regulation of the MAPK, an effector of Raf/MEK/ERK signaling pathway and a target gene of the miR-422a, leads to suppression of CRC cells proliferation. In a different study, Hu *et al*.[30]. showed that the MPAK1 down-regulation results in the inhibition of gastric cancer migration and proliferation. Considering these results, one can hypothesize that miR-6726 can potentially regulate tumorigenesis of the CRC by targeting the MAPK1. Furthermore, Li *et al*.[32] have pointed out that the RHOT1, a novel member of Rho family, induces proliferation and migration of pancreatic cancer cells. This finding suggests that the miR-614 can inhibit the tumorigenesis of tumor cells via targeting the RHOT1. It has also been shown by Liu *et al*.[33] that the miR-410 up-regulation stimulates proliferation and inhibits apoptosis of the CRC cells through targeting the BAK1 gene. Similarly, the results of Gu *et al*.[34] are indicative of the miR-150 inhibiting apoptosis and inducing proliferation of non-small cell lung cancer through targeting the BAK1. It can be concluded that the miR-7111-5p can induce the tumorigenesis of the CRC cells via targeting the BAK1. Finally, it has been displayed by Carberry *et al*.[35] that the expression level of the BOK, the pro-apoptotic gene, decreased in colorectal tumors compared to normal tissue. Another study performed by Llambi *et al*.[36] have revealed that the BOK induces apoptosis in tumor cells independently of the BAX and the BAK. In other words, the miR-1247-3p can inhibit apoptosis of colorectal tumor cells through targeting the BOK.

As a part of future work, the experimental studies should be conducted for validation of the predicted target genes of these miRNAs. For this purpose, the evaluation of four miRNAs/target genes in either clinical case control studies or *in vitro* studies will be considered. Other studies have examined miRNA in the context of CRC screening. Yamada *et al*.[37] have found different expression levels of miR-21, miR-29a, and miR-125b between healthy controls and patients with early stage CRC, with AUCs (ranging from 0.7 to 0.85) for these miRNAs. Training cohort studies by Imaoka *et al*.[38] showed circulatory miR-1290 levels to be significantly increased in patients with CRC; corresponding AUC for this miRNA was 0.830. According to the results reported by Wang *et al*.[39], the levels of circulatory miR-601 and miR-760 were significantly lower in CRC patients than in healthy controls, with AUC of the diagnostic test for miR-601 and miR-760 reported to be 0.792. Finally, Yong *et al*.[40] have demonstrated that circulatory miR-193a-3p, miR-23a, and miR-338-5p could be used for the early diagnosis of CRC by AUC 0.8. Taken together, these results and those from similar studies suggest that AUCs should be lower than 0.9 for most diagnostic tests employing circulatory miRNAs. The constructed ANN model with 4-7-1 network framework used in our study could accurately classify sample data with a better AUC[41-45]. The AUC of the designed ANN model was 1, indicating that this ANN model accurately predicts the expected results.

This research used the microarray expression of circulatory miRNAs retrieved from GEO database. The levels of circulatory miRNAs expression of this GEO dataset belong to the Japanese population. In order to generalize these finding to other populations, additional evaluation of the panel of four miRNA expression levels will be required.

In conclusion, by combining S/N and miRNA score, it was possible to identify a minimum and optimum number of miRNA biomarkers for subsequent classification of healthy and CRC samples. Based on circulating miRNA expression values, a trained ANN model accurately classified sample data into cancerous and non-cancerous categories. The precision of CRC prediction by the model was better than frequently reported methods described in the literature. As a result, by using ANN, circulatory miRNAs can be used as a non-invasive, sensitive and specific diagnostic test with potential use in CRC screening.
